# Evaluation of the Acoustic Coordinated Reset (CR®) Neuromodulation Therapy for Tinnitus: Update on Findings and Conclusions

**DOI:** 10.3389/fpsyg.2017.01893

**Published:** 2017-11-07

**Authors:** Markus Haller, Deborah A. Hall

**Affiliations:** ^1^Desyncra Technologies Limited, London, United Kingdom; ^2^National Institute for Health Research Nottingham Biomedical Research Centre, Nottingham, United Kingdom; ^3^Otology and Hearing Group, Division of Clinical Neuroscience, School of Medicine, University of Nottingham, Nottingham, United Kingdom

**Keywords:** coordinated reset, neuromodulation, RESET2, clinical trial, tinnitus

## Background

The therapeutic effects of Coordinated Reset (CR®) Neuromodulation were originally discovered by researchers at the Forschungzentrum Juelich GmbH (FZJ) in Germany, under the supervision of Tass et al. (2012a).

Based on confirmatory research in primates, initial results of Prof. Tass' research suggested that the plastic modification of the behavior of neuron populations by induced desynchronization (i.e., a process of “unlearning” of both pathological neuronal synchrony and pathological synaptic connectivity) results in a substantial and long-lasting reduction of disease symptoms (Tass et al., 2012b; Adamchic et al., 2014a).

In this set of experiments, Parkinson's-induced monkey models were treated in independent laboratories with Coordinated Reset (CR®) Neuromodulation through the well-established deep brain stimulation (DBS) technique. Standard high frequency DBS was used as a control condition. The stimulation lasted 2 h a day for 5 consecutive days. In both experiments the primates remained symptom-free for over 30 days after CR stimulation was ceased (Tass et al., 2012b; Wang et al., 2016).

Subsequent trials in human patients showed alleviation of Parkinson's symptoms such as tremor, akinesia, dystonia, etc., for many hours after only short periods of CR® Neuromodulation (4 h for 3 consecutive days) (Adamchic et al., 2014a).

These results led to an expansion and further development of the CR® Neuromodulation program in different research centers worldwide. Additional focus was given to the application of CR® via other sensory inputs, e.g., acoustic, tactile, visual, etc. That eventually led to the development of a treatment program for tinnitus.

There is a general consensus that the percept of tinnitus is triggered by cochlear damage, which degrades the auditory input to central neural pathways. This initiates physiological and electrical changes within the auditory areas that result in aberrant patterns of neuronal activity interpreted as sound.

Acoustic CR® Neuromodulation is designed as a patient specific targeted sound therapy. The treatment has been provided to over 3,000 patients worldwide to date. Several peer-reviewed papers have reported reduction of tinnitus symptoms (from baseline) in populations of tinnitus sufferers by using a portable acoustic neurostimulator providing Acoustic CR® Neuromodulation; including tinnitus loudness measured using a visual analog scale (Tass et al., 2012a; Williams et al., 2015), tinnitus annoyance measured using a visual analog scale (Tass et al., 2012a; Bencsik et al., 2015; Williams et al., 2015), and various multi-attribute scales used to measure tinnitus symptom severity, i.e., Tinnitus Questionnaire (Tass et al., 2012a), Tinnitus Handicap Questionnaire (Williams et al., 2015), and Tinnitus Handicap Inventory (Bencsik et al., 2015; Hauptmann et al., 2015).

Both accurate tone “location” of the tinnitus percept on the auditory cortex, as well as the timing and sequence of the tone based impulses have been reported to be important factors in effective inducement of neuronal desynchronization. In the studies referred to above, such neural desynchronization led to a reduction of tinnitus symptoms. Because accurate tone location in the auditory cortex was found to be important, a proprietary tinnitus pitch matching procedure was developed and deployed in order to calibrate the patient specific sound therapy tones.

Because tinnitus is a highly subjective pathology, it has been proven to be difficult to assess with objective and standardized tests. In these circumstances, any placebo effects can have a great impact on treatment of tinnitus, as well as its personal percept.

In October 2011, Nottingham University Hospitals NHS Trust agreed to conduct an investigator-led evaluation of acoustic CR neuromodulation versus placebo effects, recruiting 100 participants with a 1:1 allocation ratio (Hoare et al., 2013) (ClinicalTrials.gov NCT01541969). Participants were adults aged ≥18 years of age, chronic subjective tinnitus for more than 3months with a minimum score of 18 points measured using the Tinnitus Handicap Inventory and a dominant tinnitus pitch corresponding to a frequency between 0.2 and 10 kHz, and with an average hearing loss no greater than 60 dB (0.5, 1, 2, and 4 kHz).

## Main trial results and potential explanation of the observations

The formal results of the RESET2 trial (ClinicalTrials.gov identifier: NCT01541969) were non-conclusive, which was not expected, considering the past and current volumes of observational data showing substantial benefit (Hoare et al., 2013).

In Tass et al. (2012a) and in a number of later publications (Hauptmann et al., 2015; Williams et al., 2015) statistically and clinically significant improvements have been reported in visual analog scales, in tinnitus loudness and annoyance scores (reduction of 53 and 49% after 12 weeks of therapy, respectively, on stimulation), and in tinnitus questionnaire results (reduction of 29% after 12 weeks of therapy) results (Adamchic et al., 2012b, 2014b; Tass et al., 2012a). Furthermore, the measured tinnitus pitch was reduced by 28.5% in the first 12 weeks of treatment (Tass et al., 2012a), and the observed tinnitus pitch change was correlated with a treatment-induced reduction of tinnitus loudness and/or annoyance and changes in oscillatory brain activity (Adamchic et al., 2012a; Tass et al., 2012a). The EEG results indicated a substantial, CR-induced reduction of tinnitus-related auditory binding in a pitch-processing network associated with the therapeutic procedure, where a readjustment of stimulation parameters was performed at each visit, provided the matched tinnitus frequency had changed (Adamchic et al., 2012a).

Whilst the RESET2 trial at NHS did not observe a significant difference between treatment and placebo in experimental conditions, all the more recent above mentioned clinical studies report significant improvements in patient populations.

Tinnitus questionnaires could have reduced responsiveness to treatment-related change when a large proportion of participants respond at baseline at either extreme of the response scale. The so-called “floor effects” (scores at the minimum of the response scale) reduce sensitivity to detecting group-level improvement, while “ceiling effects” (scores at the maximum of the response scale) reduce sensitivity to detecting group-level worsening (Terwee et al., 2007). In a more recent analysis of the baseline TFI (Tinnitus Functional Index; Meikle et al., 2012) data from RESET2, Fackrell et al. (2016) found over 50% of questionnaire items scored at either floor or ceiling. This evidence certainly warrants caution in future studies with similar use of these tinnitus assessment questionnaires.

The exact application as defined of the proprietary tinnitus pitch matching procedure is paramount in order to observe any beneficial effect. Sub-optimal pitch matching will potentially lead to incorrectly computed patient specific therapy tones, which may not have any more effect than a placebo. The study-specific pitch matching procedure used in RESET2 differed from manufacturer's recommendations (Hall et al., 2016).

Professor Tass' team has since validated and launched an automated adaptive pitch-matching method (Hauptmann et al., 2016) to prevent deviations from a recommended pitch-matching procedure, which has since been utilized in all commercial applications of Acoustic CR® as well as in current and future clinical trials. The authors concluded that this new procedure offers more guidance to the audiologist and patient which is seen as of particular importance for a uniform and standardized application of pitch-matching in clinical trials. Such standardization is expected to improve the quality of measures in tinnitus therapy effectiveness.

It is reasonable to question whether only 12-weeks of device usage is sufficient for the benefit of therapy to accrue. It is noted that more recent clinical studies report alleviation of tinnitus after 6 and 12 months of therapy (Hauptmann et al., 2015; Williams et al., 2015), and data on tinnitus status at 3-, 6-, and 12-months post-fitting indicate a gradual decline in mean self-reported tinnitus severity measured using a shortened version of the Tinnitus Handicap Inventory (Hauptmann et al., 2015). The RESET2 trial design does not permit anything more than mere speculation, because all participants exited the RCT at 3 months and were unblinded to the intervention.

## Conclusions

### Recommendation

The authors therefore consider that controlled trials to test clinical effectiveness of Acoustic CR Neuromodulation for tinnitus are worthwhile. With the knowledge gained from the RESET2 trial, it is suggested that future trials should include a placebo or “usual standard of care” control group that is well characterized, should follow a well-defined and trained pitch matching protocol, should assess the status of tinnitus for longer than 12 weeks (we suggest at least 6 months), should better control the baseline characteristics to avoid floor and ceiling effects and should use an outcome instrument with known measurement properties for the target population.

### What else could be learned from the RESET2 trial?

Among the many other aspects learned another key fact was demonstrated by the RESET2 results:

During the trial 50 patients received stimulation from tones delivered at frequencies very different from the tinnitus frequency perceived by the patient and were therefore independent of used pitch-matching procedure. These stimulating tones were intended to be placebo control tones, as the trial was designed to be a double blind trial. The frequency of these tones was very different from the frequency of the tones which would have been deployed had these patients been receiving Acoustic CR® Neuromodulation treatment. Only a small “placebo-” benefit, i.e., <7.5% of an improvement in THQ (Tinnitus Handicap Questionnaire) was observed in these patients receiving the placebo treatments.

## Ethics, consent, and permissions

Permission to conduct the study was granted by the National Research Ethics Service (NRES) Committee, East Midlands–Nottingham 1, Nottingham, UK. Written informed consent was obtained from each participant in accordance with the permissions granted. For further details see Hoare et al. (2013).

## Consent for publication

For details see Hoare et al. (2013).

## Availability of data and material

See ClinicalTrials.gov NCT01541969.

## Author contributions

Both authors read and approved the final manuscript. DH planned and conducted the trial. MH and DH performed analysis and discussed the results. Both authors drafted the manuscript and were involved in final editing.

### Conflict of interest statement

DH was awarded industry grants from The Tinnitus Clinic (Brook Henderson Group, Reading, UK), and Adaptive Neuromodulation GmbH (ANM, Köln, Germany) to conduct this trial. The views expressed are those of the author(s) and not necessarily those of the NHS, the NIHR or the Department of Health. MH is CEO of Desyncra Technologies Limited, a company of the Brook Henderson Group, who was involved in funding the RESET2 trial. The reviewer, AV, and handling Editor declared their shared affiliation.

## Abbreviations

CR®: coordinated reset
DBS: deep brain stimulation
TFI: Tinnitus Functional Index.
